# Causal interpretation of correlational studies – Analysis of medical news on the website of the official journal for German physicians

**DOI:** 10.1371/journal.pone.0196833

**Published:** 2018-05-03

**Authors:** Susanne Buhse, Anne Christin Rahn, Merle Bock, Ingrid Mühlhauser

**Affiliations:** 1 Universität Hamburg, Unit of Health Sciences and Education, Hamburg, Germany; 2 Universitätsklinikum Hamburg-Eppendorf (UKE), Institut für Neuroimmunologie und Multiple Sklerose (INIMS) Hamburg, Germany; University of Alabama at Birmingham School of Health Professions, UNITED STATES

## Abstract

**Background:**

Media frequently draws inappropriate causal statements from observational studies. We analyzed the reporting of study results in the Medical News section of the German medical journal *Deutsches Ärzteblatt* (DÄ).

**Methods:**

Study design: Retrospective quantitative content analysis of randomly selected news reports and related original journal articles and press releases.

A medical news report was selected if headlines comprised at least two linked variables. Two raters independently categorized the headline and text of each news report, conclusions of the abstract and full text of the related journal article, and the press release. The assessment instrument comprised five categories from ‘neutral’ to ‘unconditionally causal’.

Outcome measures: degree of matching between 1) news headlines and conclusions of the journal article, 2) headlines and text of news reports, 3) text and conclusions, and 4) headlines and press releases. We analyzed whether news headlines rated as unconditionally causal based on randomized controlled trials (RCTs).

**Results:**

One-thousand eighty-seven medical news reports were published between April 2015 and May 2016. The final random sample comprised 176 news reports and 100 related press releases.

Degree of matching: 1) 45% (79/176) for news headlines and journal article conclusions, 2) 55% (97/176) for headlines and text, 3) 53% (93/176) for text and conclusions, and 4) 41% (41/100) for headlines and press releases. Exaggerations were found in 45% (80/176) of the headlines compared to the conclusions of the related journal article. Sixty-five of 137 unconditionally causal statements of the news headlines were phrased more weakly in the subsequent news text body. Only 52 of 137 headlines (38%) categorized as unconditionally causal reported RCTs.

**Conclusion:**

Reporting of medical news in the DÄ medical journal is misleading. Most headlines that imply causal associations were not based on RCTs. Medical journalists should follow standards of reporting scientific study results.

## Introduction

Media regularly report on health research results. Since randomized controlled trials (RCTs) are the gold standard for questions about benefits and harms of medical interventions, it would be expected that results from RCTs with patient-relevant endpoints would be reported in the media more frequently than observational studies. However, studies with lower methodological quality are cited just as frequently [1–3]. The manner in which the information is presented is as important as which studies are selected for reporting. For instance, an analysis of news articles on vitamin D supplements between 2009 and 2014 revealed a one-sided reporting that highlighted its positive effects, which was therefore not an adequate representation of scientific findings [4]. How study results are presented in press releases and in the popular press has been analyzed internationally [2, 5–9]. In the media, results from animal studies are often extrapolated to humans, despite a lack of evidence [5, 9–11]. The clinical relevance of surrogate parameters is usually not discussed. Results from observational studies are given causal interpretations, even though they only support correlations (at the most) [5, 7, 9].

The causal interpretation of observational studies can lead to wrong medical decisions. For example, hormone replacement therapy was recommended based on observational studies of postmenopausal women [12].

In a press release in 2012, the German Network for Evidence-based Medicine (DNEbM) denounced the distorted representations and causal interpretations of association studies in the German media coverage, and called for critical assessment of scientific results by medical journalists [13].

In Germany, the Deutsches Ärzteblatt (DÄ) is the official journal of the German Medical Association and the medical journal with the largest circulation. In addition to its weekly journal, medical news reports about current study results are regularly published on the DÄ website [14]. News reports published by DÄ also contain causal wording. The aim of this work was therefore to systematically analyze the medical news from DÄ to determine the causal interpretation of study results.

## Materials and methods

A part of this work was carried out within the framework of a master thesis by the coauthor MB. The methodological approach was defined a priori [15].

### Study design

A retrospective quantitative content analysis of randomly selected medical news from the DÄ website, as well as of the corresponding original studies and press releases, was carried out.

### Sample

DÄ is the official publication of the German Medical Association (*Bundesärztekammer*) and the National Association of Statutory Health Insurance Physicians (*Kassenärztliche Bundesvereinigung* [KBV]). In 2016, the DÄ journal published 44 issues with a total circulation of 357,102 printed journals. DÄ is targeted to all physicians and medical disciplines: employed, non-employed, and retired, as well as physicians who work in non-medical areas. DÄ is one of the most widely read German medical journals, and the DÄ website had about 1.6 million visitors per month between June 2015 and May 2016 [16].

The medical news section on the DÄ website contains reports on current studies from medicine, psychology, and health [14]. The reports are linked to the abstract of the corresponding study and, if available, to its press release. About 1,000 medical news reports are published annually.

The random sampling for our study began on 1 April 2016 and included medical news reports (available back to 23 April 2015) and news announcements (from the starting date until 15 May 2016). We planned to analyze about 20% percent of the included reports. All medical news reports were saved along with their corresponding studies and press releases. Random sampling was performed using a computer-assisted random generator. Reports about interventions or epidemiological evaluations and their links to health were included. For this, the news report headline was required to have at least two linked variables, such as "Vitamin C could protect against cataracts” [17]. News about health policy topics, pathophysiological concepts, diagnostic tests, or results from prognostic studies were excluded.

### Development and pilot testing of the assessment tool

A systematic literature search identified a tool that has been used to evaluate causality statements in peer reviewed papers, press releases, and health-related news stories [5, 7]. It includes a 7-point scale ranging from "no statement" to "unconditionally causal". The instrument was translated into German and pilot-tested by a student and four researchers (one medical doctor and three nurses) of the Unit of Health Sciences and Education of the University of Hamburg. Five datasets, each consisting of the headline and text body of the medical news from DÄ, the abstract and conclusion of the original study publication, and the corresponding press release, were evaluated by the experts using the tool. Subsequently, the ratings were discussed and the evaluation tool was revised. The adapted tool comprises five categories, from „neutral”to „unconditionally causal”(Table 1).

**Table 1:** Categories of causality statements.

Category	Signal words	
	English	German
1- Neutral wording		
2- Association/correlation	Associated, linked to	Assoziiert
3- Conditional causal	Might, could, may, possibly	Könnte
4- Can cause	Can, are able to	Kann
5- Unconditionally causal	No modal verb	

### Outcome measures

The endpoint was the degree of matching between: a) the categorized headline of the medical news reports and the conclusions of the original study publication; b) the headline and the text body of the medical news report; c) the text body and the conclusions of the original studies; and d) the headline and the press release. In addition, whether the news report headlines rated as causal, based on RCTs, was analyzed.

### Data collection

A master student (MB) and a health scientist (SB) independently categorized the headline and text body of each news report, the conclusions (abstract, full text) of the original study publication, and the related full press releases (headline plus text). In some cases, statements within the text body of news reports as well as within the press releases were inconsistent, i.e. the text started with a causal statement, which was subsequently phrased more weakly. In this case, the text was classified within the weaker category. We used a similar procedure for statement inconsistencies between conclusions of the abstract and the full text of the study publications. We compared the ratings from the first 15 datasets to discuss possible discrepancies arising from the evaluation tool at an early stage. As a 100% degree of matching was obtained for these 15 datasets, the remaining datasets were categorized without any further intermediate adjustments.

To classify the study design, three categories were defined: 1) non-identifiable study design; 2) studies that allow causal interpretations (RCTs and systematic reviews/meta-analyses of RCTs); and 3) studies that at the most allow associations (such as cohort studies and case-control studies). If the study design was not reported in the abstract, the full text of the study publication was considered.

Headlines were additionally categorized by a third rater (AR). Discussions about rating differences were held to reach a consensus; if no consensus was reached by the raters, members of the pilot group were consulted. At the request of the journal, we additionally categorized the press release headlines and the first two sentences separately from the full press releases.

### Inter-rater reliability and data analysis

The inter-rater reliability (IRR) between the three raters for the headlines is given by Fleiss' kappa, and the IRR between two of the raters of the remaining entire datasets, by Cohen’s kappa. The IRR for the headlines was very good for the two raters who analyzed the entire dataset (κ = 0.95). A substantial agreement for headlines was also shown with the third rater (κ = 0.69). The lower IRR resulted from a misunderstanding about an item in the evaluation tool, which could be resolved by consensus discussions. The mean IRR for the news text bodies, study conclusions, and press releases was κ = 0.97.

The analysis of the categorized datasets with respect to the result parameters was descriptive. Absolute numbers and percentages are reported.

## Results

**Fig 1:**
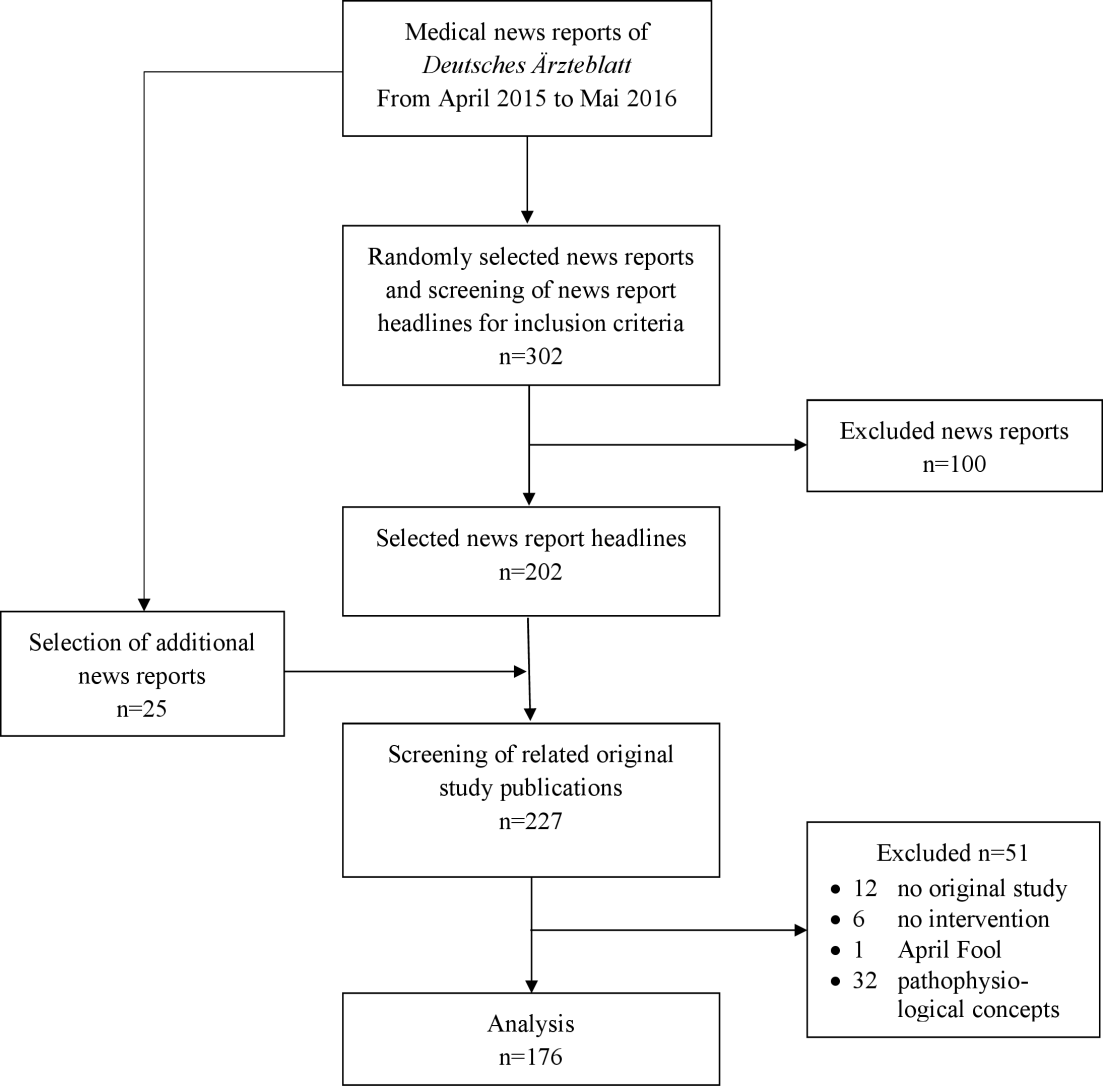
Flow chart of selected medical news reports.

From April 2015 to May 2016, 1087 medical news reports were published by DÄ. We randomly selected 302 headlines to test for inclusion criteria, of which 202 met the criteria. However, as some news reports either were not based on original studies or reported pathophysiological analyses, we included an additional 25 headlines that were randomly chosen from the remaining 785 medical news reports. Thus, we analyzed a total of 176 headlines (16%) and their related original studies. Of these studies, 100 had a corresponding press release (Fig 1).

Table 2 shows the frequency of ratings for medical news report headlines, text bodies, study conclusions, and press releases.

**Table 2:** Rating frequencies of statements.

Category	Medical news report headline	Text of medical news reports	Conclusions of the original study publication	Press releases
1 - Neutral	8	4	0	13
2 - Association	6	25	64	21
3 - Conditional causal	17	43	32	25
4 - Can cause	8	28	8	4
5 - Unconditionally causal	137	76	72	37
**Total**	176	176	176	100

Table 3 shows an example from each of the five categories.

**Table 3:** Examples of statement categorization.

Example	Material source	Material (statements)	Categories
**I**	**Medical news report headline**	*“Frisches Obst schützt vor Herz-Kreislauf-Erkrankungen und Tod”* (Fresh fruit prevents cardiovascular diseases and death) [1]*	5
	**Text of medical news report**	*“Diese Personen sind jedoch auch in anderen Bereichen … privilegiert*, *so dass bezweifelt werden kann*, *dass allein der Obstverzehr für die protektive Assoziation verantwortlich ist”* (Those people, however, are privileged in other aspects as well so that it is doubtful that fruit consumption alone is the reason for the protective association) [1]	2
	Conclusions of the original study publication	*“Among Chinese adults*, *a higher level of fruit consumption was associated with* *lower blood pressure and blood glucose levels and*, *largely independent of these* *and other dietary and nondietary factors*, *with significantly lower risks of major* *cardiovascular diseases*.*”* [2]	2
	**Press release**	*“Fresh fruit associated with lower risk of heart attack and stroke”* [3]	2
**II**	**Medical news report headline**	*“Diät kann Hypertonie nach Gestationsdiabetes vorbeugen”* (Diet can help prevent hypertension after gestational diabetes) [4]	4
	**Text of medical news report**	*“Eine gesunde Ernährung kann Frauen*, *die während einer Schwangerschaft an einem Gestationsdiabetes erkrankt sind*, *häufig vor einer arteriellen Hypertonie schützen…”* (A healthy diet can often protect women who experienced gestational diabetes during pregnancy from arterial hypertension) [4]	4
	**Conclusions of the original study publication**	*“Adherence to a healthful dietary pattern was related to a lower subsequent risk of developing hypertension among women with a history of gestational diabetes mellitus”* [5]	2
	**Press release**	*“Sticking to a healthy diet in the years after pregnancy may reduce the risk of high blood pressure among women who had pregnancy-related (gestational) diabetes…”* [6]	3
**III**	**Medical news report headline**	*“Stillen könnte Mittelohrentzündungen vorbeugen”* (Breastfeeding could prevent middle ear infections) [7]	3
	**Text of medical news report**	*“Eine Studie in Pediatrics (Online) führt dies unter anderem auf eine geringere Empfänglichkeit für respiratorische Infektionen zurück*, *wobei ein längeres Stillen der Säuglinge eine protektive Wirkung haben könnte”* (A study in *Pediatrics* (online) based this on, among other things, a lower susceptibility to respiratory infections, although prolonged breastfeeding of the infants could have a protective effect) [7]	3
	**Conclusions of the original study publication**	*“Prolonged breastfeeding was associated with significant reductions of both URI and AOM”* [8]	2
	**Press release**	*“Breastfeeding*, *a decrease in smoking and the use of new bacterial and flu vaccines have helped reduce the incidence of ear infections in babies age 12 months or younger in recent years*, *according to a new study”* [9]	5
**IV**	**Medical news report headline**	*“England*: *Mehr Geburtskomplikationen am Wochenende”* (England: More birth complications on the weekend) [10]	2
	**Text of medical news report**	*“Der Unterschied mag gering erscheinen*. *Er bedeutet aber*, *dass pro Jahr in England schätzungsweise 770 Kinder (95-Prozent-Konfidenzintervall*: *720–830) sterben*, *weil sie am Wochenende geboren wurden”* (The difference may seem small. However, it means that about 770 children (95% confidence interval: 720–830) die each year in England because they were born at the weekend) [10]	5
	**Conclusions of the original study publication**	*“…health outcomes for mothers and babies are likely to continue to be influenced by the day of delivery”* [11]	3
	**Press release**	*“Births at the weekend associated with higher rate of complications”* [12]	2
**V**	**Medical news report headline**	*“Studie untersucht Gedächtnisverlust durch Statine”* (Study investigates memory loss by statins) [13]	1
	**Text of medical news report**	*“Auch hier war die Assoziation statistisch signifikant”* (Here, too, the association was statistically significant) [13]	2
	**Conclusions of the original study publication**	*“Both statin and nonstatin LLDs were strongly associated with acute memory loss in the first 30 days following exposure in users compared with nonusers but not when compared with each other*” [14]	2
	**Press release**	*“Study examines association between cholesterol-lowering drugs*, *memory impairment”* [15]	1

*The reference list is available in: S1 Text. Reference list of Table 3.

### Comparison: News report headlines and conclusions of the original study publications

In total, 79 of 176 headlines and related original study publications were assigned to the same category. In other words, the news headline statements were consistent with the conclusions of the related study in 45% of the cases. Eight of the 176 news headlines were worded neutrally (Table 2). Exaggerations were identified in 80 headlines (45%), and nine headlines (5%) were worded weaker than the wording used by the authors of the original study publications (Table 4).

**Table 4:** Matching of ratings.

Ratings		Ratings (n)		
Medical news report headlines	Category	Conclusions of the original study publication	Text of medical news reports	Full press releases
Category: 5 Unconditionally causal (n = 137)	1	0	1	11
	2	47	19	18
	3	18	23	14
	4	5	22	3
	5	67	72	32
Category: 4 Can cause (n = 8)	1	0	0	1
	2	4	1	1
	3	0	1	1
	4	2	5	0
	5	2	1	2
Category: 3 Conditionally causal (n = 17)	1	0	0	0
	2	6	0	0
	3	8	16	7
	4	1	0	1
	5	2	1	2
Category: 2 Association (n = 6)	1	0	1	0
	2	2	2	1
	3	4	2	2
	4	0	0	0
	5	0	1	1
Category: 1 Neutral (n = 8)	1	0	2	1
	2	5	3	1
	3	2	1	1
	4	0	1	0
	5	1	1	0
Total (N)		176	176	100

### Comparison: Headlines and text of news reports

The categorized headlines and the corresponding text body of the medical news matched in 97 of 176 cases (55%). Two headlines and the corresponding texts were formulated neutrally. In a relevant proportion, the statements of the remaining headlines were weakened in the text body (68/176). Of the 137 headlines that were clearly stated in a causal manner, the corresponding text likewise contained a causal statement in 72 cases and contained a weaker formulated statement in 65 cases (Table 4).

### Comparison: Text of news reports and conclusions of the original study publications

The text of the news reports and the original study conclusions received the same rating in 93 out of 176 cases (53%). Four news reports were rated as neutral. A total of 76 news reports were identified as unconditionally causal (Table 2). From the corresponding studies of these 76 reports, 51 were also unconditionally causal, while 16 were formulated as an association. A total of 54 out of 176 news reports (31%) were exaggerated as compared to the study conclusions. In 25 out of 176 cases (14%), the study conclusions were rated higher than the wording in the text of the news report.

### Comparison: News report headlines and corresponding press releases

The categories of headlines and full press releases matched in 41% of the cases (41/100). Of the 100 news headlines that had a corresponding press release, 78 were rated as unconditionally causal. Likewise, 32 press releases (41%) were rated as unconditionally causal, 11 as neutral, 18 as association, 14 as "could" statements, and 3 as "can" statements (Table 4, S1 Table).

The degree of matching between the text body of the medical news and the press release was 46% (data not shown).

The additional comparisons of DÄ headlines with press release headlines showed a matching of 49% (49/100); DÄ headlines and press release headlines plus the first two sentences matched in 43% (43/100) of the cases (S1 Table).

### Comparison: News report headlines and study design

A total of 137 out of 176 headlines were rated as unconditionally causal (Table 2). However, only 52 studies (38%) described the results of RCTs, while 85 (62%) described cohort studies, case-control studies, or reviews of studies with low evidence levels.

## Discussion

Only about half of the medical news reports on the DÄ website agree with the conclusions of the original study publications. The news headlines often contain causal statements, even though the majority of them only describe observational studies. This leads to study results being presented in the medical news reports in an exaggerated and therefore misleading manner.

### Strengths and limitations

A representative sample from the medical news reports of DÄ was analyzed. The randomly selected news reports contained publications with a broad range of health topics, ranging over a year. To categorize the medical news reports as well as the original study publications, a previously developed tool was used that has already been applied several times [5, 7]. For our study, the original seven categories were reduced to five. One reason for this was due to translation and thus understandability of the categories. For example, the German translations of the signal words "associated with" and "linked to" were interpreted to be the same in the pretest. Putting these terms into separate categories, as in the original tool, therefore did not make sense for either the IRRs or the analysis. The applicability of the adapted instrument was ensured by the pilot and the pretest. Categorization of the datasets was performed independently by two raters with a very good inter-rater reliability. Nevertheless, there remains some degree of subjectivity. The categorization of the headlines by a third rater showed more disagreements, which was largely caused by a missing test phase with the third rater and a misunderstood item in the assessment tool. However, this highlighted the fact that some of the headlines had a greater scope for interpretation. The headline “Coffee drinkers live longer” was categorized as unconditionally causal by all three raters. Later in the process of summarizing the results, it was interpreted as an associative statement by one of the authors (IM). We presented the headline as an example for an unconditional causal statement to physicians and other health professionals at two scientific conferences. Our rating was not questioned. However, we checked again all of the included headlines regarding this kind of statements. No more comparable headlines were identified. Further, headlines containing the modal verb "can" also allowed several interpretations. In these cases, both the categories of “can cause” and “unconditionally causal” are possible, if “can” is understood as "can actually". To ensure transparency, our ratings of the datasets are available in the Supplementary Information (S2 Table).

As we did not present the news reports to the target group of DÄ, we do not know how physicians interpret the headlines.

We did not carry out a critical evaluation of RCTs. In fact, RCTs with a poor methodological quality might not allow a causal interpretation of the results. Therefore, the proportion of headlines with causal statements that were correctly based on valid RCTs might be still too high in our analysis. Furthermore, we did not check the correctness of the conclusions of the original study publications. Study conclusions often do not correspond to the study results. For instance, one study of articles reporting RCTs with non-significant results found that the conclusions given in both the abstract and in the text body distorted the results in more than a third of the cases [18]. In order to determine formulation and interpretation of study results in the news section on the DÄ website, we performed a descriptive analysis of data. We refrained from additional inferential statistics. It remains unclear if proportions are different from chance or not. However, results are in line with our assumptions.

### Significance of results

This work confirms the results of previous investigations. Similar to media reports and press releases [1–3], the medical news reports of DÄ do not mainly report large RCTs with patient-relevant endpoints but rather results from observational studies.

The rate of exaggeration through causal interpretation of association studies is also confirmed in the literature. In their analysis, Sumner *et al*. found that 40% of university press releases contain recommendations that are not confirmed in the original publication [5]. This was also found to be the case for 36% of news reports (print and online). In one-third of the press releases and in 39% of news reports, association studies were interpreted causally. Exaggerations in press releases were associated with exaggerations in the corresponding news reports [5]. In another study, the same group examined media reports and journal press releases [7]. In this case, the press releases contained slightly fewer exaggerations but still contained recommendations and causal interpretations in 23% and 21% of cases, respectively. As in the previous work, a link between exaggerations in press releases and news reports could be found [7].

Qualitatively better press releases seem to be linked with more transparent media reports [6]. If a press release mentioned the harms of an intervention, absolute numbers, or study limitations, the related newspaper articles also mentioned these more frequently [6].

The medical news reports of DÄ showed only a weak correlation with the corresponding press releases. In contrast to Sumner et al. [5, 7], we categorized the full press release rather than only headlines and the first two sentences in our main analyses. We deliberately decided not to categorize the headline and text of the press releases separately, in the first place. We expect medical journalists to read the full press release and not only the headline. We even expect medical journalists to check the original study before writing the news report. However, the categorization of the press release headlines plus the first two sentences showed similar results as the analysis of the full press releases. Our study did not address the quality of press releases.

An investigation of press releases, media reports, and RCTs showed that media reports mainly contain the same misinterpretations as press releases [8]. The same misinterpretation was also found in the abstracts of the RCT publications. “Spinning” the results in the press releases (by overly stressing certain positive aspects) was likewise associated with a spin in the study abstracts [8].

Such distortions have an influence on the interpretations of study results. For instance, in an RCT performed with clinicians, Boutron *et al*. first correctly reformulated 30 abstracts from RCTs for which the original publication exaggerated a non-significant primary endpoint [19]. For the study, 300 clinicians then received either the original abstracts or the reworded correct versions. The participants rated the original abstracts with the distortion to show higher benefits from the interventions than the rewritten versions, and they were more interested in reading the original abstracts than the reworded (non-exaggerated) ones [19].

The extent to which the type of coverage in the medical news reports of DÄ determines whether a physician would like to read the corresponding original study publication has so far not been investigated. The influence on professional activity thus remains unclear.

However, media can influence the health-related behavior of both physicians and citizens [20–22]. The wide dissemination of the results from the Women's Health Initiative study in the media is seen as a factor for the rapid reduction of the number of women with hormone replacement therapy [23]. In a Danish prospective cohort study, the early discontinuation of statins was associated with negative reporting about statins. Conversely, positive reporting was associated with continued statin use [24].

### Conclusions

Our results show that standards for reporting results from scientific work are not taken sufficiently into account in the medical news reports of DÄ. The main focus of the news section should not be to draw attention but rather to send the correct message. A neutral phrasing / wording of the headlines could prevent misinterpretations, which possibly also result from translation (from the original language into German). Further, mentioning the study design in the headline would be preferable, similar to the required reporting standards for titles of study publications [25, 26]. For example, a neutral wording of the headline “Fresh fruit prevents cardiovascular diseases and death” could be: “Cohort study examines the association between fruit consumption and cardiovascular events”.

For readers, it is not clear who is writing the medical news reports. Thus, crediting the authors of the reports, as a basic prerequisite for transparency and responsibility, should be standard in the DÄ news section.

Medical journalists have a difficult role, especially when press releases and abstracts of the studies are distorted. Critical evaluation of studies requires a corresponding competence. The German Network for Evidence-based Medicine (DNEbM) regularly offers workshops for journalists.

## Supporting information

S1 Text Reference list of Table 3. (DOCX)

S1 Table Categorization of press releases. (PDF)

S2 Table Ratings of all medical news reports, study conclusions, and press releases. (XLSX)
