# Supplementary material for: Causal interpretation of correlational studies – Analysis of medical news on the website of the official journal for German physicians
Source: PLoS One. 2018 May 3;13(5):e0196833. doi: 10.1371/journal.pone.0196833 (PMC5933791; doi:10.1371/journal.pone.0196833)
Supplement: S1 Text — (DOCX) [file pone.0196833.s001.docx]

**S1 Text. Reference list of Table 3**

1. Deutsches Ärzteblatt: Frisches Obst schützt vor Herz-Kreislauf-Erkrankungen und Tod. 2016. Available from: https://www.aerzteblatt.de/nachrichten/66248/Frisches-Obst-schuetzt-vor-Herz-Kreislauf-Erkrankungen-und-Tod.

2. Du H, Li L, Bennett D, Guo Y, Key TJ, Bian Z, et al.: Fresh Fruit Consumption and Major Cardiovascular Disease in China. NEJM. 2016; 374: 1332-43.

3. University of Oxford: Fresh fruit associated with lower risk of heart attack and stroke. 2016. Available from: http://www.ox.ac.uk/news/2016-04-06-fresh-fruit-associated-lower-risk-heart-attack-and-stroke.

4. Deutsches Ärzteblatt: Diät kann Hypertonie nach Gestationsdiabetes vorbeugen. 2016. Available from: https://www.aerzteblatt.de/nachrichten/66368.

5. Li S, Zhu Y, Chavarro JE, Bao W, Tobias DK, Ley SH, et al.: Healthful dietary patterns and the risk of hypertension among women with a history of gestational diabetes mellitus: a prospective cohort study. Hypertension. 2016; 67: 1157-65.

6. National Institute of Child Health and Human Development: Healthy diet may reduce high blood pressure risk after gestational diabetes, NIH study suggests. 2016. Available from: https://www.nichd.nih.gov/news/releases/Pages/041816-healthy-diet.aspx.

7. Deutsches Ärzteblatt: Stillen könnte Mittelohrentzündungen vorbeugen. 2016. Available from: https://www.aerzteblatt.de/nachrichten/66150.

8. Chonmaitree T, Trujillo R, Jennings K, Alvarez-Fernandez P, Patel JA, Loeffelholz MJ, et al.: Acute Otitis Media and Other Complications of Viral Respiratory Infection. Pediatrics. 2016; 137 (4): doi: 10.1542/peds.2015-3555.

9. American Academy of Pediatrics: Ear Infections Among Infants Decline: Research Credits New Vaccines, Breastfeeding and Lower Smoking Rates. 2016. Available from: https://www.aap.org/en-us/about-the-aap/aap-press-room/Pages/Ear-Infections-Among-Infants-Decline-Research-Credits-New-Vaccines-Breastfeeding-and-Lower-Smoking-Rates.aspx.

10. Deutsches Ärzteblatt: England: Mehr Geburtskomplikationen am Wochenende. 2015. Available from: https://www.aerzteblatt.de/nachrichten/64929/England-Mehr-Geburtskomplikationen-am-Wochenende.

11. Palmer WL, Bottle A, Aylin P: Association between day of delivery and obstetric outcomes: observational study. BMJ. 2015; 351: h5774.

12. Imperial College of London: Births at the weekend associated with higher rate of complications. 2015. Available from: http://www3.imperial.ac.uk/newsandeventspggrp/imperialcollege/newssummary/news_24-11-2015-16-14-49.

13. Deutsches Ärzteblatt: Studie untersucht Gedächtnisverlust durch Statine. 2015. Available from: https://www.aerzteblatt.de/nachrichten/63067/Studie-untersucht-Gedaechtnisverlust-durch-Statine.

14. Strom BL, Schinnar R, Karlawish J, Hennessy S, Teal V, Bilker WB: Statin Therapy and Risk of Acute Memory Impairment. JAMA Intern Med. 2015; 175: 1399-405.

15. Journals TJN: Study examines association between cholesterol-lowering drugs, memory impairment. 2015. Available from: https://www.eurekalert.org/pub_releases/2015-06/tjnj-sea060415.php.
