# Supplementary material for: Causal interpretation of correlational studies – Analysis of medical news on the website of the official journal for German physicians
Source: PLoS One. 2018 May 3;13(5):e0196833. doi: 10.1371/journal.pone.0196833 (PMC5933791; doi:10.1371/journal.pone.0196833)
Supplement: S1 Table — (PDF) [file pone.0196833.s002.pdf]

S1 Table. Categorization of press releases

Ratings		Ratings (n)		
**Medical news report headlines with PR**	**Category**	**PR headlines**	**PR headlines plus first two sentences**	**Full PR**
Category: 5 Unconditionally causal (n = 78)	1	9	11	11
	2	10	14	18
	3	11	14	14
	4	3	2	3
	5	45	37	32
Category: 4 Can cause (n = 5)	1	1	1	1
	2	1	1	1
	3	1	1	1
	4	0	0	0
	5	2	2	2
Category: 3 Conditionally causal (n = 10)	1	0	0	0
	2	0	0	0
	3	3	5	7
	4	0	1	1
	5	7	4	2
Category: 2 Association (n = 4)	1	0	0	0
	2	0	0	1
	3	2	2	2
	4	0	0	0
	5	2	2	1
Category: 1 Neutral (n = 3)	1	1	1	1
	2	1	1	1
	3	1	1	1
	4	0	0	0
	5	0	0	0
Total (N=100)		100	100	100

PR=press release
